# Messages for ultraviolet‐radiation protection to fair‐skinned populations

**DOI:** 10.1111/jdv.70148

**Published:** 2025-11-06

**Authors:** Lieve Brochez, Claus Garbe, Teresa Amaral, Petr Arenberger, Violeta Astratinei, Philippe Autier, Marianne Berwick, Matilda Bylaite, Brigitte Boonen, Veronique del Marmol, Brigitte Dreno, Maria Concetta Fargnoli, Adele C. Green, Rüdiger Greinert, Axel Hauschild, Catherine A. Harwood, Isabelle Hoorens, Lidija Kandolf, Roland Kaufmann, Nicole Kelleners‐Smeets, Aimilios Lallas, Celeste Lebbé, Ulrike Leiter, Henry W. Lim, Caterina Longo, Joseph Malvehy, David Moreno, Fredrik Östman, Giovanni Pellacani, Ketty Peris, Caroline Robert, Bettina Ryll, Philippe Saiag, Dirk Schadendorf, Peter Soyer, Gilliosa Spurrier‐Bernard, Eggert Stockfleth, Alex Stratigos, Hisashi Uhara, Ricardo Vieira, Martin A. Weinstock, Dagmar Whitaker, David C. Whiteman, Iris Zalaudek, Ana‐Maria Forsea, EADO Task Force for Prevention and Early Detection of Skin Cancer

**Affiliations:** ^1^ Department of Dermatology Ghent University Hospital Ghent Belgium; ^2^ Centre for Dermatooncology, Department of Dermatology Eberhard Karls University Tuebingen Germany; ^3^ Department of Dermatovenereology Charles University Third Medical Faculty and University Hospital Kralovske Vinohrady Prague Czech Republic; ^4^ Melanoma Patient Network Europe Uppsala Sweden; ^5^ International Prevention Research Institute (i‐PRI) Dardilly France; ^6^ University of New Mexico Comprehensive Cancer Centre Albuquerque New Mexico USA; ^7^ Faculty of Medicine, Centre of Dermatovenereology, Clinic of Infectious Diseases and Dermatovenereology Vilnius University Vilnius Lithuania; ^8^ Belgian Cancer Foundation Brussels Belgium; ^9^ Department of Dermatology, Erasme Hospital Université Libre de Bruxelles Brussels Belgium; ^10^ France Nantes University, Inserm 1302, INCIT Nantes France; ^11^ San Gallicano Dermatological Institute IRCCS Rome Italy; ^12^ Population Health Department QIMR Berghofer Medical Research Institute Brisbane Queensland Australia; ^13^ Skin Cancer Centre, Laboratory for Molecular Cell Biology Elbe Hospital Buxtehude Buxtehude Germany; ^14^ Department of Dermatology University Hospital Schleswig‐Holstein (UKSH) Kiel Germany; ^15^ Department of Dermatology, Barts Health NHS Trust and Centre for Cell Biology and Cutaneous Research Blizard Institute, Barts and the London School of Medicine and Dentistry Queen Mary University London UK; ^16^ Department of Dermatology, Faculty of Medicine Military Medical Academy Belgrade Serbia; ^17^ Department of Dermatology, Venerology and Allergology Frankfurt University Hospital Frankfurt Germany; ^18^ sGROW School for Oncology and Reproduction Maastricht University Medical Centre Maastricht The Netherlands; ^19^ Department of Dermatology Maastricht UMC+ Comprehensive Cancer Centre Maastricht The Netherlands; ^20^ First Department of Dermatology, School of Medicine, Faculty of Health Sciences Aristotle University Thessaloniki Greece; ^21^ Université Paris Cite, AP‐HP Dermato‐Oncology and CIC, Cancer Institute APHP, Nord Paris Cité Paris France; ^22^ Department of Dermatology Henry Ford Health Detroit Michigan USA; ^23^ Zienda Unità Sanitaria Locale – IRCCS di Reggio Emilia Skin Cancer Centre Reggio Emilia Italy; ^24^ Department of Dermatology, Hospital Clínic de Barcelona (Melanoma Unit) University of Barcelona, IDIBAPS, Barcelona & CIBERER Barcelona Spain; ^25^ abMedical‐&‐Surgical Dermatology Service Hospital Universitario Virgen Macarena Sevilla Spain; ^26^ Department of Clinical Internal, Anesthesiological and Cardiovascular Sciences Sapienza University Rome Italy; ^27^ Dermatologia, Dipartimento di Medicina e Chirurgia Traslazionale Università Cattolica del Sacro Cuore Rome Italy; ^28^ UOC di Dermatologia, Dipartimento di Scienze Mediche e Chirurgiche Fondazione Policlinico Universitario A. Gemelli – IRCCS Rome Italy; ^29^ Department of Medical Oncology Gustave Roussy and Paris Saclay University Villejuif France; ^30^ Department of General and Oncologic Dermatology, Ambroise Paré Hospital, APHP, & EA 4340 “Biomarkers in Cancerology and Haematooncology”, UVSQ Université Paris‐Saclay Boulogne‐Billancourt France; ^31^ Department of Dermatology & West German Cancer Centre University Hospital Essen & German Cancer Consortium & National Centre for Tumour Diseases (NCT)‐West Essen Germany; ^32^ Dermatology Research Centre, Frazer Institute The University of Queensland Brisbane Queensland Australia; ^33^ Department of Dermatology Ruhr‐University Bochum Germany; ^34^ First Department of Dermatology University of Athens School of Medicine, Andreas Sygros Hospital Athens Greece; ^35^ Department of Dermatology Sapporo Medical University Sapporo Japan; ^36^ Department of Dermatology and Venereology Centro Hospitalar Universitário de Coimbra Coimbra Portugal; ^37^ Brown University and V A Medical Centre Providence Rhode Island USA; ^38^ Private Practice Cape Town South Africa; ^39^ Department of Dermatology and Venereology of the Hospital Clinics Giuliano Isontino (ASUGI) Maggiore Hospital Trieste Italy; ^40^ Dermatology Department, Elias University Hospital Carol Davila University of Medicine and Pharmacy Bucharest Romania

**Keywords:** educational materials, melanoma, patient education as topic, photoprotection, skin neoplasms/prevention & control, ultraviolet rays/adverse effects

## Abstract

**Background:**

Skin cancer prevention remains a critical public health challenge, particularly in fair‐skinned populations in Europe, the United States and Australia, where incidence rates of keratinocyte skin cancer and melanoma continue to rise despite decades of public education on ultraviolet‐radiation (UVR) protection. Although progress has been observed in Oceania, the overall effectiveness of current prevention strategies remains insufficient. This paper aims to refine and disseminate more effective UVR protection messages by developing an evidence‐based, internationally adaptable public education leaflet.

**Methods:**

The development of this educational material followed the current guidelines for the development of health promotion materials and effective public education. Based on the scientific evidence, a plain‐language message has been drafted. It was subsequently revised through multiple rounds of multi‐stakeholder feedback from dermato‐oncology, epidemiology, public health experts, patient organizations representatives and NGOs involved in prevention and health promotion.

**Results:**

The final educational leaflet emphasizes three core messages: avoiding intentional sun exposure and tanning, utilizing shade and protective clothing as primary UV protection strategies and using sunscreen as a supplementary protective measure. Additional recommendations address childhood sun protection, the dangers of tanning beds and the importance of monitoring skin for early signs of cancer. Common concerns such as vitamin D synthesis and sunscreen safety are also addressed with evidence‐based responses.

**Discussion:**

This initiative highlights the necessity of shifting public attitudes towards UVR exposure and developing tailored, culturally sensitive communication strategies. The freely available leaflet will be distributed through professional associations and online platforms. Future efforts should involve policymakers in implementing structural changes, such as enhancing public shade availability and regulating tanning facilities, to promote long‐term behavioural shifts in UV protection.

## Graphical abstract

This expert consensus summarizes messages for UV protection in fair‐skinned populations. Key messages include no sunbathing or tanning, no sunbeds. UV protection measures are to be used from UV index 3. Priority is given to shade and physical protection. Sunscreen with a strong SPF is to be used on uncovered areas.

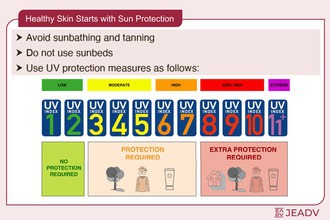

**Why was the study undertaken?**

- This paper aims to develop an evidence‐based, internationally adaptable public education leaflet to improve the effectiveness and dissemination of UVR protection messages.

**What does this study add?**

- The educational leaflet highlights three key messages: refrain from intentional sun exposure and tanning; prioritize shade and protective clothing as the main forms of UV protection; and use sunscreen as a complementary measure.

**What are the implications of this study?**

- This initiative underscores the need to shift public attitudes on UVR exposure through tailored, culturally sensitive communication strategies. By distributing this leaflet via professional channels and online, the authors wish to facilitate this broader change. Long‐term impact also requires policy‐level action such as increasing public shade and regulating tanning salons.

## INTRODUCTION

For decades, dermatologists and other physicians have been educating their fair‐skinned patients about protecting themselves from ultraviolet radiation (UVR) in order to avoid skin cancer.^1^ This applies especially to fair‐skinned populations whose individuals are particularly sensitive to the development of skin cancer. The use of sunscreens has been a central part of this education.

Despite ongoing patient and public education, there has not yet been a decrease in the incidence rates of keratinocyte skin cancer or melanoma.^2^, ^3^, ^4^ This is particularly true for Europe and the United States. In Oceania, however, a flattening and incipient fall in incidence rates is evident, although incidence rates in Australia and New Zealand are still much higher than in Europe or the United States.^5^

Despite ongoing patient education and numerous public awareness campaigns, the goal of primary prevention of skin cancer in Europe and the USA has not yet been achieved. Rather, we are seeing a sustained increase in incidence rates, unlike any other cancer entity.

This raises the question of whether we are really conveying the right messages for effective UV protection, and what hurdles we have to take into account when implementing these messages. We have already discussed the right messages in an article with authors from five continents based on available scientific evidence. In this process, we have changed the central messages. The focus is now on avoiding sunbathing and tanning, protecting from UVR with clothing and seeking shade, and only in third place is the use of sunscreen.^6^

There are a number of hurdles to implementing the message about sun protection. For decades, sun holidays have been associated with the intention of tanning, and tanning has been seen as healthy.^7^ There are reservations about UV protection, such as the idea that vitamin D synthesis may become insufficient.^8^, ^9^ Many underestimate the risk of invisible UVR exposure and disregard the far later risk of developing skin cancer. Even knowing about the risk of UVR is not immediately translated into behaviour.^10^ In addition, using sunscreen can be quite costly.

The aim of this project is to develop an information leaflet (core text) for educating the patients and the public about the correct measures of UV protection. It will summarize the most important messages on this topic in a clear and concise way. It is aimed to be the core message that can be afterwards taken up and integrated into any public campaign of education for skin cancer prevention. It is planned to translate it into many languages and distribute it through national and international professional associations.

## METHODS

The methodology of development of the public education material builds on the theoretical basis of the development of health promotion materials,^11^ and follows the current guidelines for developing efficient patient and public health education tools.^12^

The informational content of the educational material was retrieved from the scientific evidence summarized in the recent global consensus publication ‘Skin cancers are the most frequent cancers in fair‐skinned populations, but we can prevent them’.^6^ We utilized a large‐scale, publicly accessible artificial intelligence language model to generate a plain‐language adaptation of a scientific text. The original material,^6^ which included evidence‐based recommendations for photoprotection, was input into the model with instructions to simplify specialized terminology and tailor the content for a non‐expert, lay public audience. The resulting draft of a public education brochure was then reviewed by an international group of dermato‐oncology experts of the EADO Cancer Prevention Task Force, in order to ensure accuracy and clarity.

The resulting draft was revised by the EADO network of Dermato‐Oncology experts (meeting in Rome, 8 Novmber 2024); the subsequent version was submitted for patients' and public feedback on readability and comprehension, clarity, actionability and relevance to the patients' representatives of the Melanoma Patients Network Europe (Chair: Bettina Ryll); the Patient Scientific Advisory Board of Ghent University Hospital (Coordinator: Wendy Van De Velde, Chair: Natacha Laeremans). Subsequently, the revised material was reviewed by the Euroskin Board (President: Lieve Brochez), comprising dermatologists, epidemiologists, public health experts, etc.

The final version of the educational text was revised by the consortium of authors of this paper.

## RESULTS

### Healthy skin starts with sun protection

One in three cancers diagnosed is a skin cancer. Exposure to ultraviolet (UV) radiation from the sun or artificial UV sources (sunbeds, solaria) is the leading cause of all skin cancer types, both melanoma (arising from pigment cells in the skin) and keratinocyte cancers (arising from the keratinocytes in the skin, including basal cell carcinoma and squamous cell carcinoma). Although getting outside when it is sunny feels enjoyable for most of us and is important for physical and mental wellbeing, protecting your skin from UV is essential to reduce your risk of developing skin cancer.

#### Sun exposure can speed up skin ageing

UV exposure is responsible for about 80% of visible signs of skin ageing. These include wrinkles, fine lines, sagging skin and age spots. Sun‐protective habits and regular use of sunscreen can help preserve youthful, healthy skin.

#### UV damage adds up over time and can be prevented

Skin damage from UV exposure builds up over time. The sun emits UV radiation, which can damage skin cells by causing DNA mutations, that is, changes in the genetic material of cells. These changes may lead to sunburn, premature ageing and an increased risk of skin cancer. Even if you don't get sunburn, your skin may still be damaged. Prevention is key, and it is never too late to start protecting your skin from further damage. This is advised for all skin types.

#### Children are especially vulnerable

Unprotected sun exposure during childhood and teenage years significantly increases the risk of developing skin cancer later in life. Encourage sun‐safe habits in children early by using hats, protective clothing and additional sunscreens on uncovered skin when outdoors.

### How to protect your skin?

The recommendations are summarized in Table 1.

**TABLE 1:** Protect your Skin Check Box.

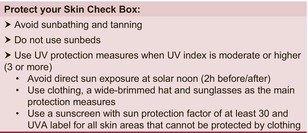

#### Avoid sunbathing

The World Health Organization classifies ultraviolet light as a proven cause of cancer. It is mainly our UV behaviour that determines the risk for skin cancer, especially our habits of sunbathing, where large parts of the skin are exposed to the sun. Using sunscreen during sunbathing does not provide adequate protection of the skin. In contrast, sunscreen use during sunbathing may even increase the risk of skin cancer, due to prolonged exposure to more intense sun.

#### Avoid tanning

There is no such thing as a ‘safe’ or ‘healthy tan’ from UV rays. A tan is an SOS signal of the skin trying to defend itself from sun damage.

#### Avoid tanning beds

Tanning beds are a significant source of UV radiation and have been proven to increase your risk for all skin cancer types from the very first exposure and especially at younger ages. Tanning beds also accelerate skin ageing.

#### Start protecting your skin when UV index is 3 or higher

**FIGURE 1:**
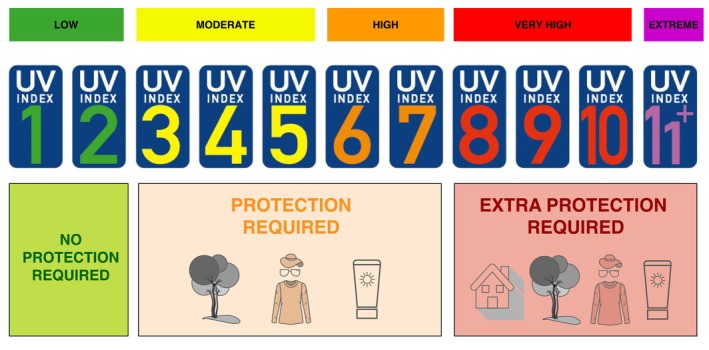
UV protection measures according to UV index. (adapted from https://cancer‐code‐europe.iarc.who.int/.)

The UV index is a measure of the strength of ultraviolet (UV) radiation from the sun at a specific place and time. The index typically ranges from 0 (minimal risk) to 11+ (extreme risk). It indicates the potential risk of harm (Figure 1).

#### Avoid the sun at midday

UV index will be highest 2 h before and after solar noon, meaning when the sun reaches its highest position in the sky. This means if you want to go outside or if you are planning outdoor activities, outside the time period of 2 h before/after solar noon are the best timings to do so. If you have no choice but to go outside around solar noon, use the protection measures as mentioned below.

#### Shade and protective clothing are your best friends

When outdoors, especially at noon, seek shade. Wear a wide‐brimmed hat, long‐sleeved shirts and trousers/skirts below the knee and sunglasses with UVA and UVB filter. Opt for tightly woven fabrics for the best protection.

#### Sunscreen is essential for uncovered skin

When UV index is 3 or more, use sunscreen with both UVA label and high UVB sun protection factor (SPF) (SPF 30 to 50+) on the skin areas that cannot be protected by clothes. Apply before sun exposure and reapply at least once a day, more often after swimming and sweating.

### Watch that SPOT!

Monitor your skin for any new growths, changing moles, or non‐healing sores. Contact your GP or a dermatologist in case of one of these signs. Early detection of skin cancer dramatically increases the chance of successful treatment.

### Frequently asked questions

#### Does consistent UV protection cause vitamin D deficiency?

The sun‐safe behaviour as described above does not usually lead to vitamin D deficiency in people with fair skin who are normally active outdoors. If too low vitamin D levels are diagnosed in your case, discuss with your GP the intake of dietary sources of vitamin D or vitamin D supplements rather than increasing ultraviolet exposure.

#### Can I prepare my skin for holidays with sunbeds?

No. Pre‐tanning is not giving a lot of protection, but instead adds to the skin damage. The use of sunbeds has been shown to increase the risk of all skin cancer types and accelerate skin ageing.

#### Am I completely UV protected when indoor or in the shade?

Not completely. Be aware that UVA rays can penetrate the windows of buildings or cars and reach your skin even indoors. UV (A and B) rays reflected from surrounding surfaces like water, sand, or concrete can also cause damage to the uncovered skin in the shade.

#### Do sunscreens contain harmful substances?

Some chemical (as opposed to mineral) UV filters can enter the blood when used in high amounts. However, commercially available sunscreens have been approved as safe for human use by regulatory authorities if used correctly, that is applied limited to skin areas that cannot be protected by clothes, together with other protective measures like shade and avoiding midday sun exposure.

Sunscreen residues can wash off into the sea, where they have been found in fish and water worldwide. At high concentrations (higher than those usually found in the sea), some UV filters can harm corals and fish reproduction. The full impact needs to be further studied.

## DISCUSSION

This information leaflet summarizes the recently published international recommendations for patients and the general population.^13^ A first draft was generated by the EADO Prevention Task Force, with AI support and revised by the EADO board, the Melanoma Patient Network Europe and other stakeholders.

The reduction of avoidable UV exposure constitutes an important pillar to manage the skin cancer epidemic.^3^ At present, one in every three cancers diagnosed is a skin cancer. Europe has the highest burden of skin cancers in terms of absolute numbers worldwide, while Oceania has the highest incidence rates. Further increases are expected for the coming decades. Exposure to UV is the dominant driver amenable to preventive actions in these increases and adequate UV protection is a cost‐saving health intervention.

The combination of seeking shade, protection by clothing and the use of sunscreens is well established.^14^ However, in real life, people tend to overestimate the protection from sunscreens. A report of the International Agency for Research on Cancer concluded that sunscreen use does not offer protection in conditions of intentional sun exposure (sunbathing and tanning). There are strong indications that this may even induce a new risk behaviour for skin cancer, especially melanoma.^15^ Overall, the reduction of intentional sun exposure is important to reach adequate UV protection in the population. This implies a change in the culture of sunbathing/tanning.

Since primary prevention of skin cancer has a positive return on investment, it is important to spread these recommendations to the broad public, to healthcare providers and to health authorities as a first step towards their implementation in daily life. To facilitate access, this information leaflet will be available and freely downloadable in different languages on the websites of EADO (https://eado.org/), EuroMelanoma (https://www.euromelanoma.eu/), EuroSkin (https://euroskin.org/) and other organizations engaged in the field of skin cancer prevention. Creating an inclusive communication that promotes behavioural changes in the general population is a challenge that may require the expertise of public health workers and community psychologists; it may be good to learn from the experiences of Australia in this field. The use of other media channels to communicate this message to the public may involve the deployment of a communication office. Adapted communication strategies may be needed in different countries and various target groups.

Finally, policymakers will need to be addressed. They have an important responsibility in communicating UV index and associated protection messages to the public, in creating adequate shade facilities in schools, recreational areas, outdoor workplaces and in a strict regulation or ban on the use of commercial tanning facilities. A study published in 2019 investigating sunbed legislation in Europe reported the absence of legislation in 25% of responding countries and around one‐third of countries had no restrictions for minors.^16^ An update on health authority initiatives in primary prevention of skin cancer would be useful to evaluate if further steps have been taken in this field, given the skin cancer pandemic. In addition, it can encourage the exchange of best practices.

## AUTHOR CONTRIBUTIONS

First, second and last author: conceptualization, original draft preparation, writing, reviewing, editing. All other authors: writing, reviewing.

## FUNDING INFORMATION

No funding was available for this work.

## CONFLICT OF INTEREST STATEMENT

LB is president of Euroskin, has received a speaker fee from EADV, ESDO, EADO and a research gift from Beiersdorf. CG has received personal fees from CeCaVa, MSD, NeraCare and Philogen. VA is affiliated with Asociatia Melanom Romania and Stichting Melanoom. BD has been a consultant for Galderma, Almirall, Fabre, NAOS, BMS, Huvy IA, Biofortis and Sanofi. MCF has participated in the advisory board of Pierre Fabre and La Roche‐Posay. IH has received a travel grant from Sunpharma. RK has received a consulting fee from Regeneron and honoraria from Leo Pharma. HL's institution has received grants from Incyte, La Roche‐Posay, Pfizer and PCORI and personal consulting fees from ISDIN, Beiersdorf, Ferndale, L'Oréal, Eli Lilly, Zerigo Health, Skinosive, Kenvue, Cantabria Labs and honoraria from La Roche‐Posay, Cantabria Labs, Pierre Fabre, NAOS, Uriage, Pfizer and ISDIN. FO is affiliated with the Swedish Melanoma Association (Melanomföreningen). DCW has received consulting fees from MelNet, Cancer Institute NSW, New Zealand Cancer Control Agency, Melanoma Institute Australia and a speaker fee from EADO. AMF's institution has received a consultancy fee from MagnaPharm/Laboratoires ACM and support for attending a meeting from La Roche‐Posay. TA, PAr, PA, MB, BB, VDL, AG, RG, AH, CH, LK, NKS, CLe, CLo, JM, DM, GP, KP, CR, BR, PSa, DS, PSo, GSB, ES, AS, HU, RV, MAW, DW, IZ have no conflicts of interest.

## ETHICAL APPROVAL

No ethical committee approval was retrieved for this work.

## ETHICS STATEMENT

This paper followed ICMJE Recommendations on patient privacy and informed consent.

## Data Availability

The data that support the findings of this study are openly available in ‘Skin cancers are the most frequent cancers in fair‐skinned populations, but we can prevent them. *Eur J Cancer*. 2024;204:114074. https://doi.org/10.1016/j.ejca.2024.114074’.
